# Development and evaluation of a machine learning based risk prediction model for osteoporosis

**DOI:** 10.3389/fmolb.2026.1939155

**Published:** 2026-09-24

**Authors:** Lin Zhou, Qi Zhang, Haijing Wei, Jie Liu, Yongchang Yang

**Affiliations:** Department of Clinical Laboratory Medicine, The Seventh Medical Center Chinese PLA General Hospital, Beijing, China

**Keywords:** biomarkers, cytokine, machine learning, osteoporosis, prediction model

## Abstract

**Objective:**

Osteoporosis is a systemic bone disease that can lead to decreased bone strength and increased susceptibility to fractures. This study aims to evaluate the level of cytokines and other common blood biomarkers, and establish a machine learning-based risk prediction model for osteoporosis patients.

**Methods:**

The clinical data of patients with osteoporosis who were hospitalized in the Seventh Medical Center of the Chinese People’s Liberation Army General Hospital from January 2023 to September 2024 were retrospectively collected. 16 cytokines and other potential predictive variables were analyzed to determine the independent risk factors. Eleven machine learning models were established, and the performance of the models was evaluated using the ROC curve, the PR curve and the confusion matrix, and the optimal model was selected. SHAP to provide insights into the model’s predictions and construct a visual representation of the prediction model.

**Results:**

A total of 187 patients were included, and 16 characteristics such as IL-8 were identified as risk characteristics. By comparing 11 different models, it was found that the Gradient Boost was the best model, with an AUC value of 0.91, an accuracy rate of 0.81, an F1 score of 0.80, and a relatively balanced specificity and sensitivity. And the SHAP value was used to reveal the direction and strength of each feature in predicting osteoporosis.

**Conclusion:**

This study successfully developed a risk prediction model based on 16 common variables, including IL-8, providing critical evidence for the early identification and prevention of osteoporosis patients, with potential clinical significance.

## Introduction

Osteoporosis is a systemic bone disease characterized by a declined in bone mineral density (BMD) and deteriorated of bone microarchitecture, which lead to decreased bone strength and increased susceptibility to fractures (Pepe et al., 2020). As a major global public health issue, its impact on people aged 50 and above is particularly severe, with a lifetime risk of fragility fractures of 50% for women and 20% for men (Tough and Batur, 2020; Anish and Nair, 2024). With the continuous acceleration of global population aging, osteoporosis has become an increasingly serious threat to public health, and its related socio-economic burden is expected to increase significantly in the coming decades (Foger-Samwald et al., 2020). At present, the majority of osteoporosis patients have not received standardized diagnostic assessments and evidence-based treatment interventions, which in turn has led to a sustained increase in global fragility fracture rates.

In clinical practice, dual energy X-ray absorptiometry (DXA) is the gold standard for evaluate BMD and confirming the diagnosis of osteoporosis (LeBoff et al., 2022; Song et al., 2022). Although DXA technology has reduced radiation exposure to safe levels, the persistent anxiety of patients towards radiation fear cannot be ignored. In addition, the reliance on professional equipment poses a significant obstacle to its widespread adoption, especially in resource limited primary healthcare settings (Anupama et al., 2025; Berry, 2018). In contrast, detection technology based on blood biomarkers has superior clinical applicability and significant advantages over traditional methods, especially in improving patient compliance and enhancing medical accessibility.

The hematological indicators of osteoporosis mainly evaluate bone metabolism, endocrine function, and other potential pathogenic factors. Furthermore, deviations in routine hematological parameters such as whole blood cell count and liver and kidney function tests may be related to the pathogenesis or progression of osteoporosis (Song et al., 2022; Ebina et al., 2025). Recently, a few researches shown that cytokines were related to the immune inflammatory responses *in vivo* through complex regulatory networks. In the pathological mechanism of osteoporosis, cytokines play a pivotal role in the dynamic balance between osteoclasts and osteoblasts (Elahmer et al., 2024; Fischer and Haffner-Luntzer, 2022).

At present, various osteoporosis risk assessment tools have been developed worldwide. among which, FRAX ® The fracture risk assessment tool is a typical example (Amarnath et al., 2023; Schini et al., 2024). This tool integrates clinical factors and BMD data to accurately estimate the probability of an individual experiencing severe osteoporotic fractures and hip fractures within 10 years (El, 2020; Lewiecki et al., 2020). However, the tool still relies on BMD data and has not yet incorporated the predictive value of blood biomarkers such as osteocalcin.

Our study conducted a comprehensive comparative analysis of multidimensional hematological features between patients with osteoporosis and matched healthy controls, with a focus on exploring the relationship between BMD measurements and hematological indicators. By integrating various hematological parameters across cellular, metabolic, and endocrine dimensions, we use advanced machine learning techniques to identify key biomarker patterns and construct an intelligent diagnostic framework for osteoporosis, providing support for clinical decision. Furthermore, by comprehensively analyzing these biomarkers, our study aims to provide effective preventive strategies for high-risk populations.

## Materials and methods

### Clinical data

This research enrolled participants who underwent DXA for osteoporosis diagnosis. Osteoporosis participants met the following inclusion criteria: (1) DXA detection bone density T-value ≤ −2.5 at lumbar spine or femoral neck; (2) A history of fragility fractures (de Souza, 2010; Sabri et al., 2023). The participants with any of the following conditions were excluded from this research: (1) A history of malignant tumors, cerebrovascular accidents, acute myocardial infarction, or other serious acute/chronic diseases; (2) Concurrent diagnosis of inflammatory disease or autoimmune diseases, etc. (3) Incomplete medical record information that precluded adequate study assessment (Siris et al., 2014; Snodgrass et al., 2022). According to the inclusion criteria, 359 participants (including osteoporosis patients and sex-and age-matched healthy controls during the same period) were recruited from the seventh Medical Center Chinese PLA General Hospital from January 2023 to September 2024. And 187 eligible participants met all standards and were involved in the ultimate analysis. This research was approved by the Ethics Committee of our institution (S2025-076-01).

Based on collecting and summarizing existing literature and clinical experience, we identified the predictor variables for this study. These variables included demographic and anthropometric parameters (age, age at menopause, height, weight, body mass index (BMI)). Additionally, 98 chief laboratory tests indicators, including blood cell counts, C-reactive protein, bilirubin, lipid profile, cytokines, and other relevant biomarkers, were retrospectively collected for all participants.

All serum samples were obtained via centrifugation after blood collection and stored at −80 °C. Serum concentrations of cytokines, including IFNα, IFNγ, IL-10, IL-17F, IL-1, IL-18, IL-22, IL-2R, IL-2, IL-17A, IL-8, IL-4, were quantified using chemiluminescence assay. The reagent kit was purchased from Orienter Bioengineering Co., Ltd (Sichuan, China), and experiments were conductedaccording to the manufacturer’s instructions.

### Statistical analysis

Normally distributed data was represented as mean ± standard deviation, and intergroup comparisons were performed by t-tests. Non-normally distributed data was represented as median and analyzed by Kruskal–Wallis H test. Missing values for each variable were count using “VIM” package in R and imputed via the “zoo” package in R. Using the createDataPartition function in the R “caret” package to split data into training and validation cohort in a 7:3 ratio. The Boruta algorithm was applied to the cohort to perform preliminary feature selection and identify key variables.

Logistic regression, AdaBoost, Decision tree, EXtreme Gradient Boosting (XGBoost), Artificial Neural Network (ANN), LightGBM, GradientBoost, K-Nearest Neighbors (KNN), Support Vector Machine (SVM), Random forest and Naïve bayes were used to screen risk factors for osteoporosis, and a novel predictive model was built according to the identified factors.

During the model construction process, L2 regularization is used to constrain the model weights and limit the excessive amplification of feature weights to reduce the risk of overfitting; At the same time, the model’s generalization performance will be objectively evaluated through an independent test set (30% samples) to avoid the problem of performance overestimation caused by relying solely on the training set for evaluation. Accuracy, positive recall, negative recall, positive precision, negative precision, F1 score, Receiver Operating Characteristic (ROC) curve and the area under the ROC curve (AUC) were calculated to evaluate the predictive performance of the model. This research used SPSS 26.0 software (SPSS Inc., United States of America) and R software (version 4.5.1, United States of America).

## Results

Firstly, 359 individuals were involved in this study. Among them, 123 were excluded because of incomplete medical record information, and 49 were excluded for meeting the predefined exclusion criteria. Ultimately, 187 participants were finally enrolled, including 125 osteoporosis patients and 62 healthy controls during the same period. The flowchart was shown in Figure 1. The demographic and clinical characteristics of all participants are summarized in Table 1.

**FIGURE 1:**
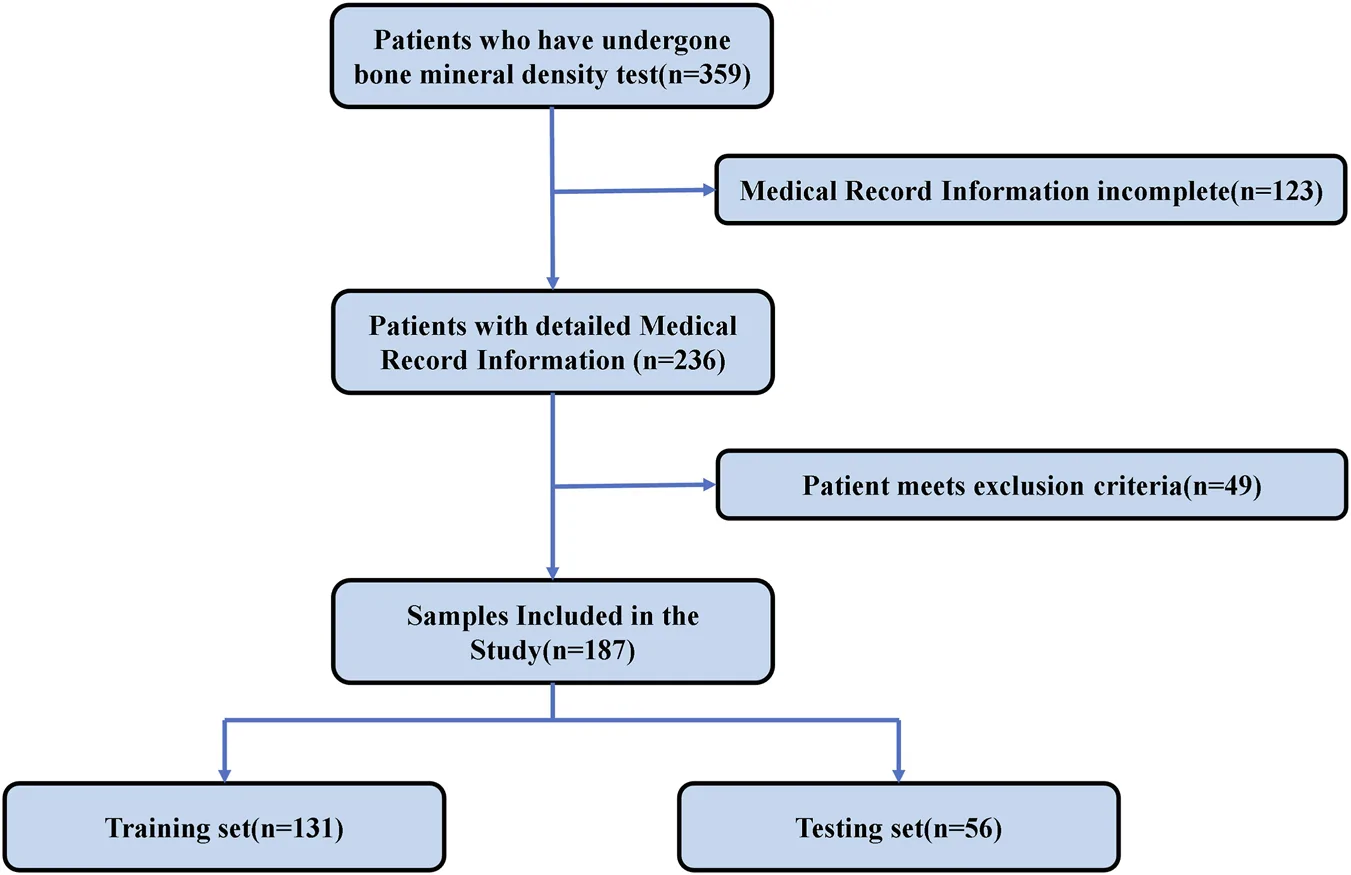
The flowchart of the enrollment.

**TABLE 1:** The demographic and clinical characteristics of the participants.

	Total	Normal	Osteoporosis	P-value
Age	61.37hight6.13	60.79 ± 6.36	61.66 ± 6.02	0.365
Age at menopause	49.76 ± 3.98	51.42 ± 3.39	48.97 ± 4.01	0.000
Height	159.60 ± 5.4	162.19 ± 4.96	158.31 ± 5.16	0.000
Weight	61.86 ± 10.21	67.44 ± 10.09	59.10 ± 9.11	0.000
BMI	24.24 ± 3.52	25.64 ± 3.69	23.55 ± 3.23	0.000
Femoral T-score	−1.80 ± 1.59	0.22 ± 0.66	−2.81 ± 0.70	0.000
Lumbar spine T-score	−1.67 ± 1.80	0.50 ± 1.11	−2.75 ± 1.01	0.000
Anemia	Yes 36	9	27	
	No 148	52	96	0.267
Diabetes	Yes 16	8	8	
	No 169	53	116	0.130

Comparison of cytokine levels between the two groups revealed that the levels of IL-8 and IL-18 were significantly decrease in the osteoporosis group (Figure 2). These results suggest that abnormalities in various cytokines may be contribute to the pathogenesis of osteoporosis.

**FIGURE 2:**
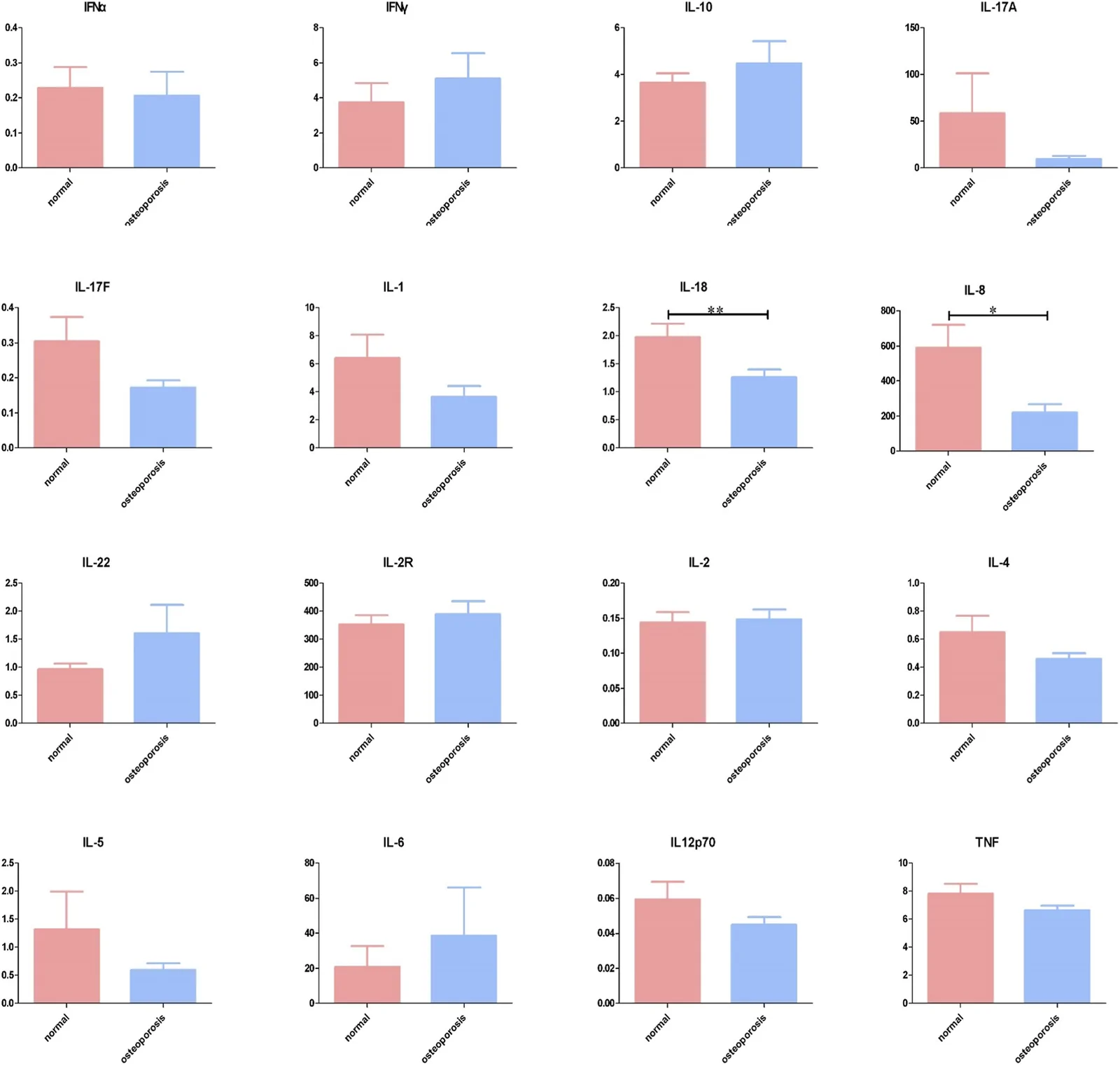
The differences in cytokines between health group and osteoporosis group (Statistical significance: *P < 0.05, **P < 0.01)

Notably, the osteoporosis group exhibited significantly lower age of menopause and BMI compared to healthy group (p < 0.001). A total of 96 potential predictive variables were included in the initial analysis. Meanwhile, missing values within each variable across all included participants were quantified, and missing data were imputed with the mean value for numerical vatiables (Figure 3a). According to univariate analysis, 24 of these features reveled significant differences between the two groups (*p* < 0.05) (Figure 3b), specifically: BGP, CTx, IL-8, PINP, D-DIMER, AFU, CHE, TG, CHO, ALP, UA, INR, PTA, PT, RBC, P-LCR, BASO%, EO#, HGB, PDW, MPV, PCT, BAP, LDL.

**FIGURE 3:**
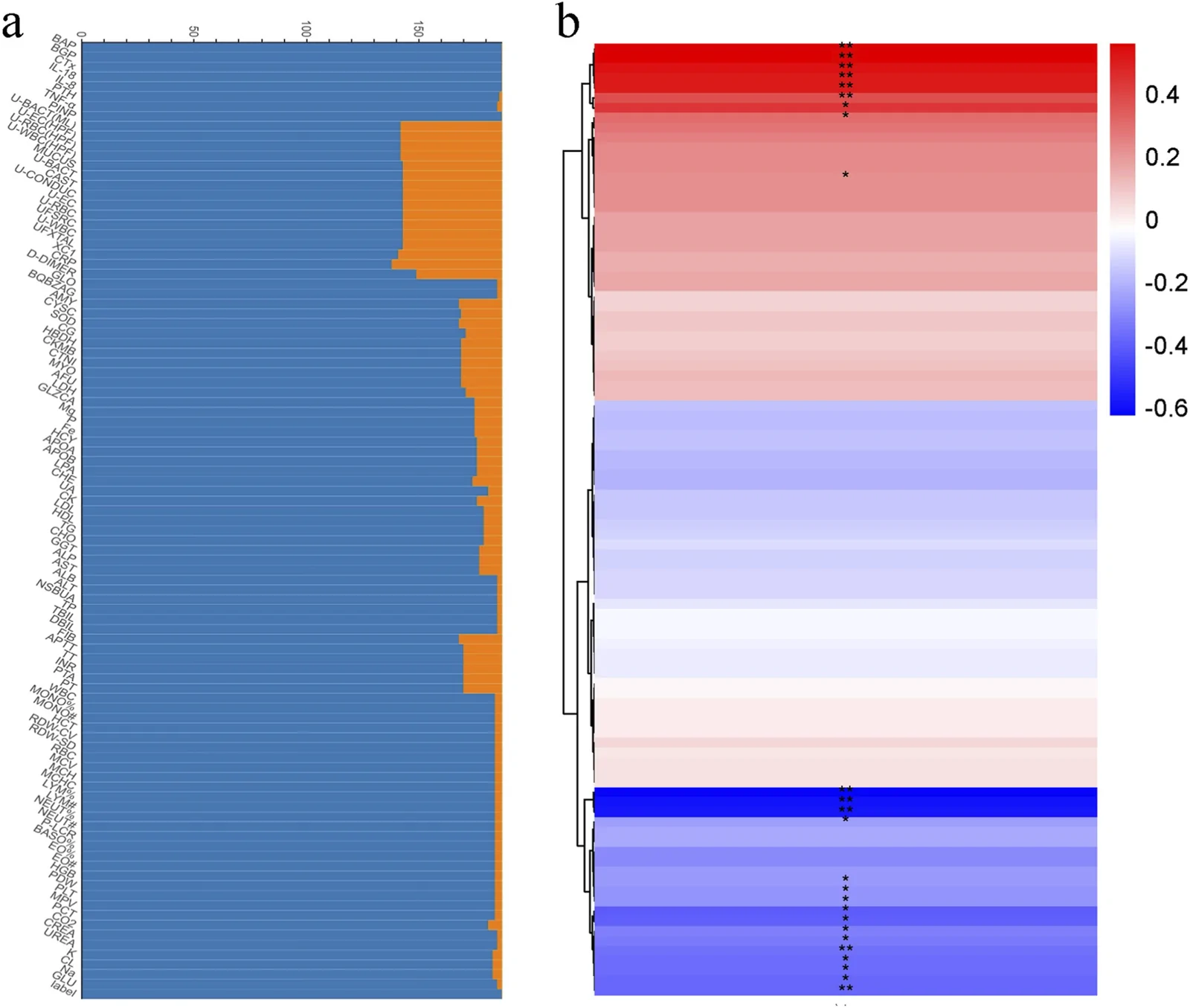
**(a)** The percentage of missing values in each feature **(b)** comparison of each feature in health participate and osteoporosis patients.

Figure 4a illustrates the correlation analysis of these features among participants, with several features showning high correlation (Figure 4a). Only one representative feature was retained from each set of highly correlated variables to avoid multicollinearity. Boruta visually displays the importance of each feature, as shown in Figure 4b. Green boxplots indicate that the corresponding variables are confirmed as important predictors, while red boxplots denote rejected variables and yellow boxplots represent tentative variables. Decision curve analysis (DCA) was applied to analysis the clinical applicability, the 16 features model and 19 features model achieved equivalent net benefits, which were superior to those of 12 features model (Figure 4c). Above all, 16 risk features, including BGP, CTx, IL-8, PINP, D-DIMER, AFU, TG, CHO, ALP, NSBUA, INR, RBC, P-LCR, PCT and BAP, were ultimately selected to construct predictive model. The expression differences of the 16 risk characteristics mentioned above are shown in Supplementary Figure S1.

**FIGURE 4:**
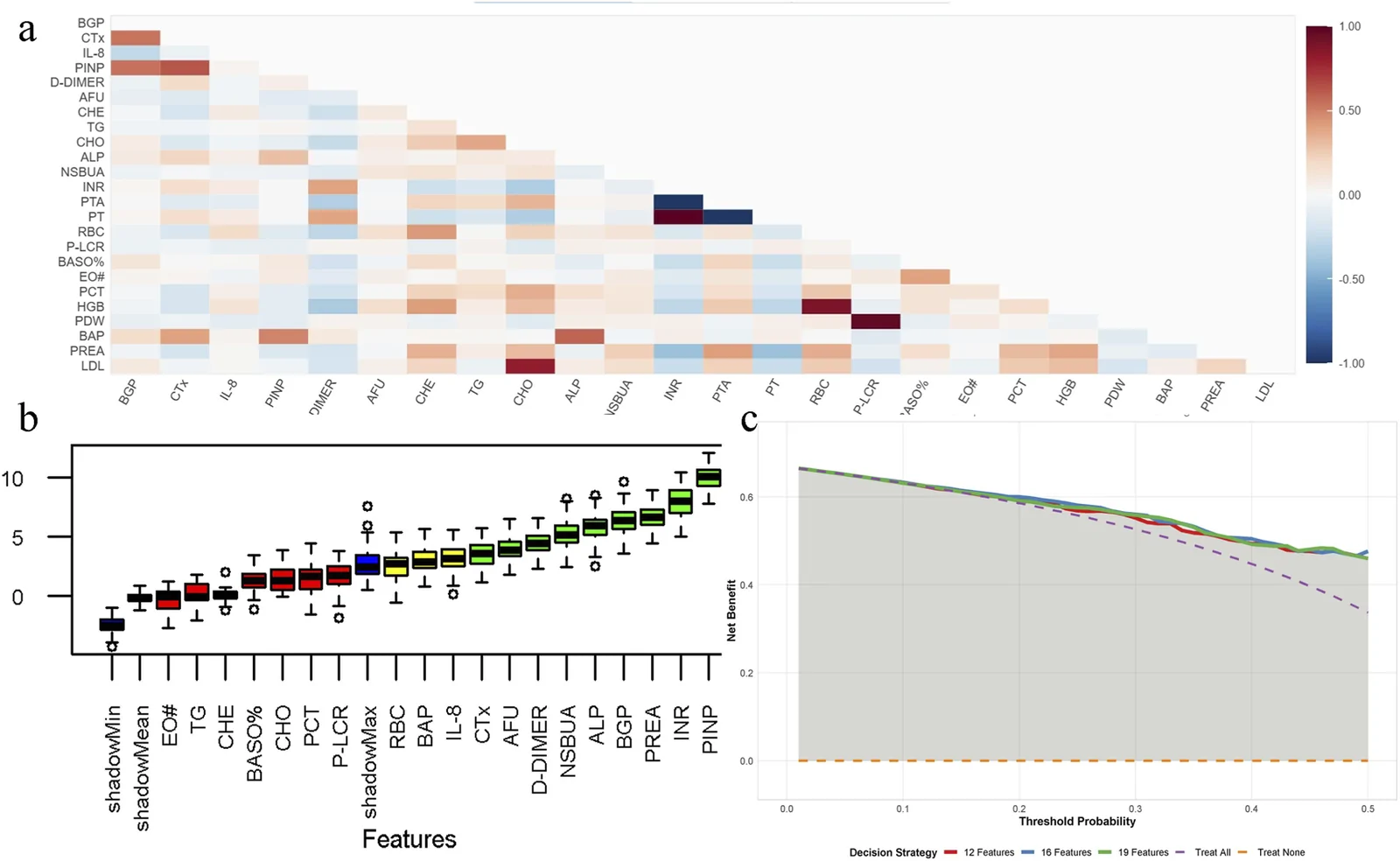
**(a)** Heatmap illustrating the correlation between each features. **(b)** Boruta boxplots to quantifying and presenting the importance of each feature. **(c)** Decision curve analysis in the three different feature models.

After data preprocessing, all data was randomly divided into training and validation cohort in a 7:3 ratio. In this study, eleven distinct machine learning algorithms were employed to establish the predictive model, including Logistic regression, AdaBoost, Decision tree, XGBoost, ANN, LightGBM, GradientBoost, KNN, SVM, Random forest and Naïve bayes. The performance of these models, which evaluated by accuracy, precision, recall, and F1 score, was reported for both the training and validation cohort in Table 2. The accuracy and AUC of multiple models in the training set of this study reached 1.0, indicating the presence of overfitting. The main reason is that the total sample size of this study was only 187 cases, and the modeling included 16 predictive features, resulting in a relatively high proportion of features compared to the sample size. However, the significant decrease in various indicators of the test set proves that the model still has stable predictive ability in independent samples and has clinical reference value. And the performance of each model is compared in multiple dimensions via the radar map to further improve visual interpretability (Figure 5).

**TABLE 2:** The comparation of eleven models.

		Accuracy	Positive recall	Negative recall	Positive precision	Negative precision	F1 score	Kappa	95% CI
Train	logistic_regression	0.71	0.85	0.42	0.75	0.58	0.69	0.29	0.67–0.84
	AdaBoost	1.00	1.00	1.00	1.00	1.00	1.00	1.00	0.99–1.00
	Decision tree	0.75	0.9	0.44	0.76	0.68	0.73	0.37	0.59–0.75
	Random forest	0.98	1.00	0.95	0.98	1.00	0.98	0.96	0.99–1.00
	XGBoost	1.00	1.00	1.00	1.00	1.00	1.00	1.00	0.99–1.00
	LightGBM	1.00	1.00	1.00	1.00	1.00	1.00	1.00	0.99–1.00
	GradientBoost	1.00	1.00	1.00	1.00	1.00	1.00	1.00	0.99–1.00
	SVM	0.97	0.99	0.93	0.97	0.98	0.97	0.93	0.99–1.00
	ANN	0.78	0.94	0.47	0.78	0.8	0.77	0.46	0.77–0.89
	naive_bayes	0.51	0.34	0.84	0.81	0.39	0.5	0.14	0.63–0.82
	KNN	0.78	0.87	0.6	0.82	0.7	0.78	0.5	0.79–0.91
Test	KNN	0.65	0.84	0.26	0.7	0.45	0.62	0.12	0.58–0.85
	Decision_ tree	0.74	0.89	0.42	0.76	0.67	0.72	0.35	0.54–0.78
	SVM	0.72	0.92	0.32	0.73	0.67	0.69	0.27	0.56–0.85
	LightGBM	0.79	0.87	0.63	0.82	0.71	0.79	0.51	0.72–0.94
	XGBoost	0.75	0.92	0.42	0.76	0.73	0.73	0.38	0.69–0.92
	GradientBoost	0.81	0.92	0.58	0.81	0.79	0.80	0.54	0.80–0.97
	logistic_regression	0.74	0.87	0.47	0.77	0.64	0.73	0.37	0.61–0.086
	random_forest	0.7	0.92	0.26	0.71	0.62	0.66	0.22	0.79–0.97
	AdaBoost	0.7	0.84	0.42	0.74	0.57	0.69	0.28	0.65–0.90
	ANN	0.67	0.87	0.26	0.7	0.5	0.63	0.15	0.52–0.80
	Naive _bayes	0.47	0.45	0.53	0.65	0.32	0.49	−0.023	0.37–0.68

**FIGURE 5:**
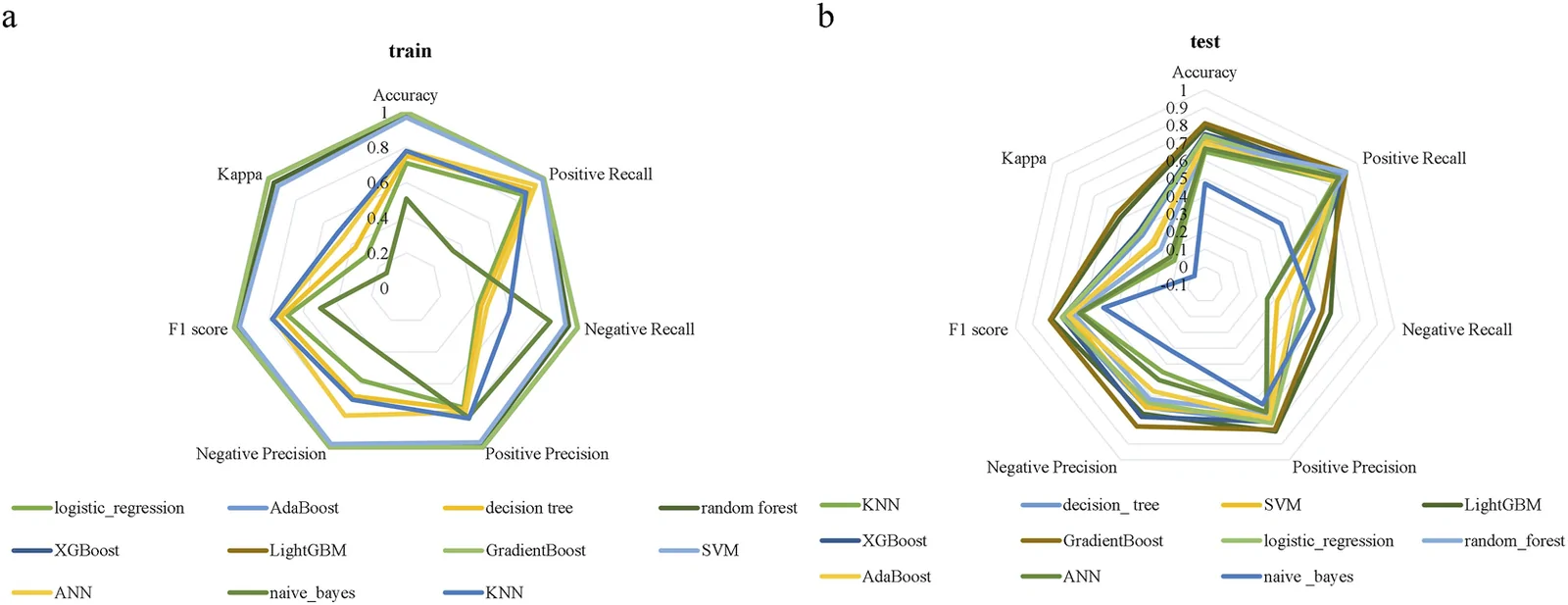
Radar map for comparative performance of each model. **(a)** Training set; **(b)** Test set.

Based on comprehensive evaluation of ROC curves and PR curves, six models demonstrated high predictive performance: Gradient boost, Random forest, LightGBM, XGBoost, Logistic regression, KNN (Figure 6).

**FIGURE 6:**
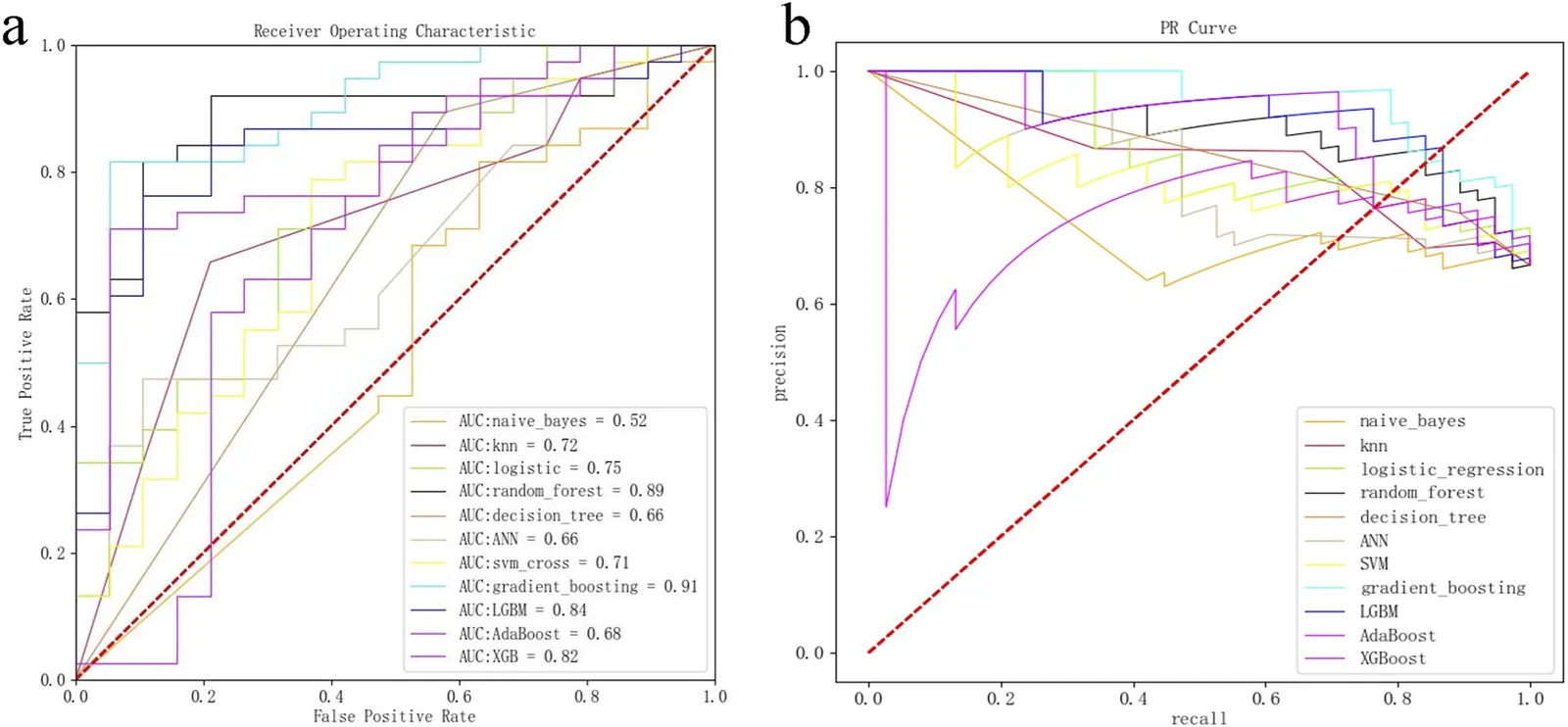
The ROC curve **(a)** and PR curve **(b)** of machine learning models.

Moreover, the Confusion Matrix for the six models are presented in Figure 7. As a fundamental visualization tool for demonstrating model performance in machine learning, confusion matrices explicitly illustrate the classification results of each model, specifically the number of true positives, true negatives, false positives and false negatives, which providing a direct reference for evaluating the models.

**FIGURE 7:**
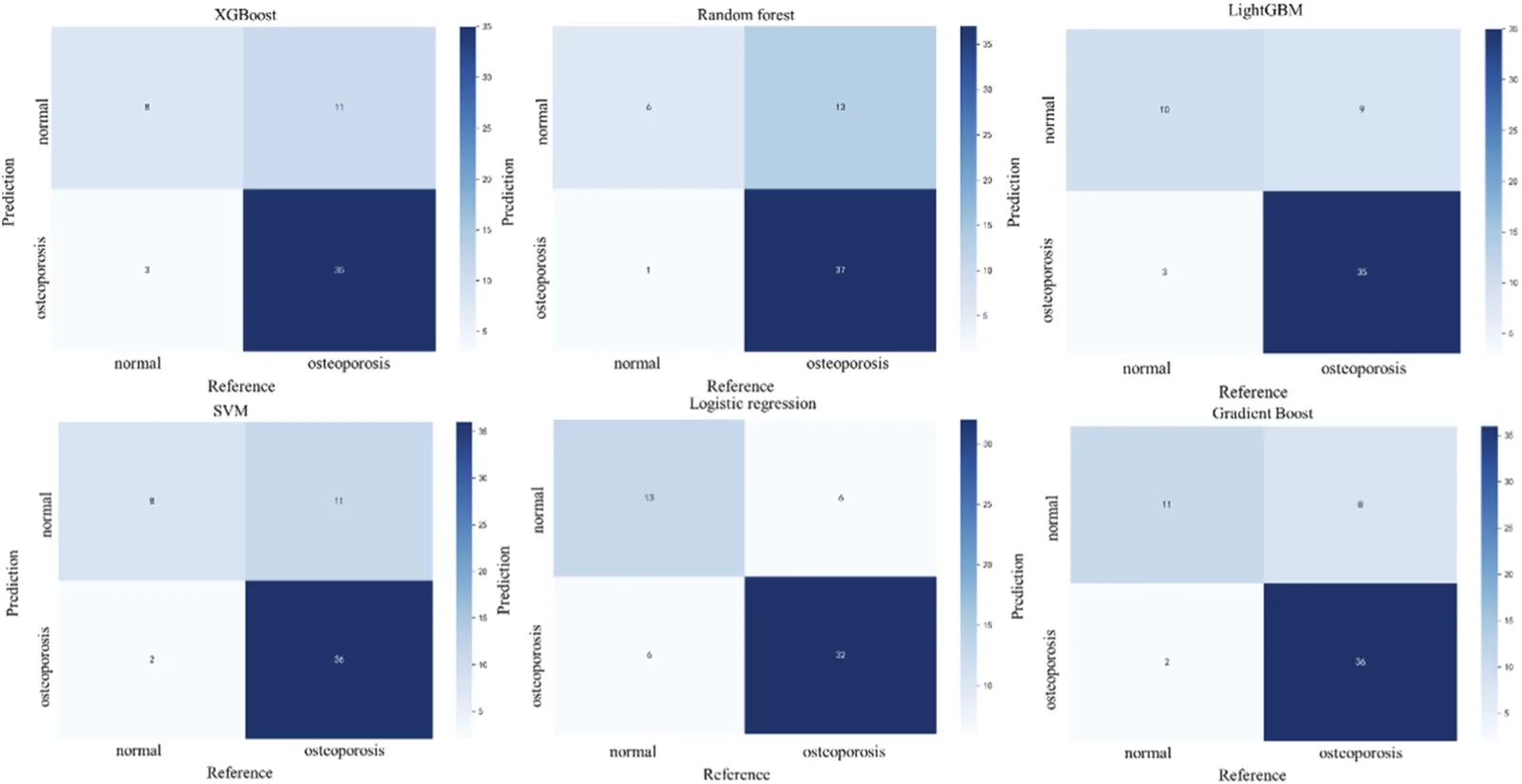
The confusion matrix of six models.

For the SHapley Additive exPlanations (SHAP) value (Figure 8a), each line shows the SHAP values for the features assigned to all participants. The x-axis represents the SHAP value, which revealed the contribution of a certain feature to the prediction result. Its absolute value reflects the intensity of features on the model’s predictive outcome. Moreover, the SHAP values of five samples were calculated by Gradient Boost model (Figure 8c), which intuitively illustrates how the model generates predictions based on these features and visualizes the interpretive logic of SHAP. A nomogram was also constructed to quantify the relative importance of these features in predicting osteoporosis (Figure 8b).

**FIGURE 8:**
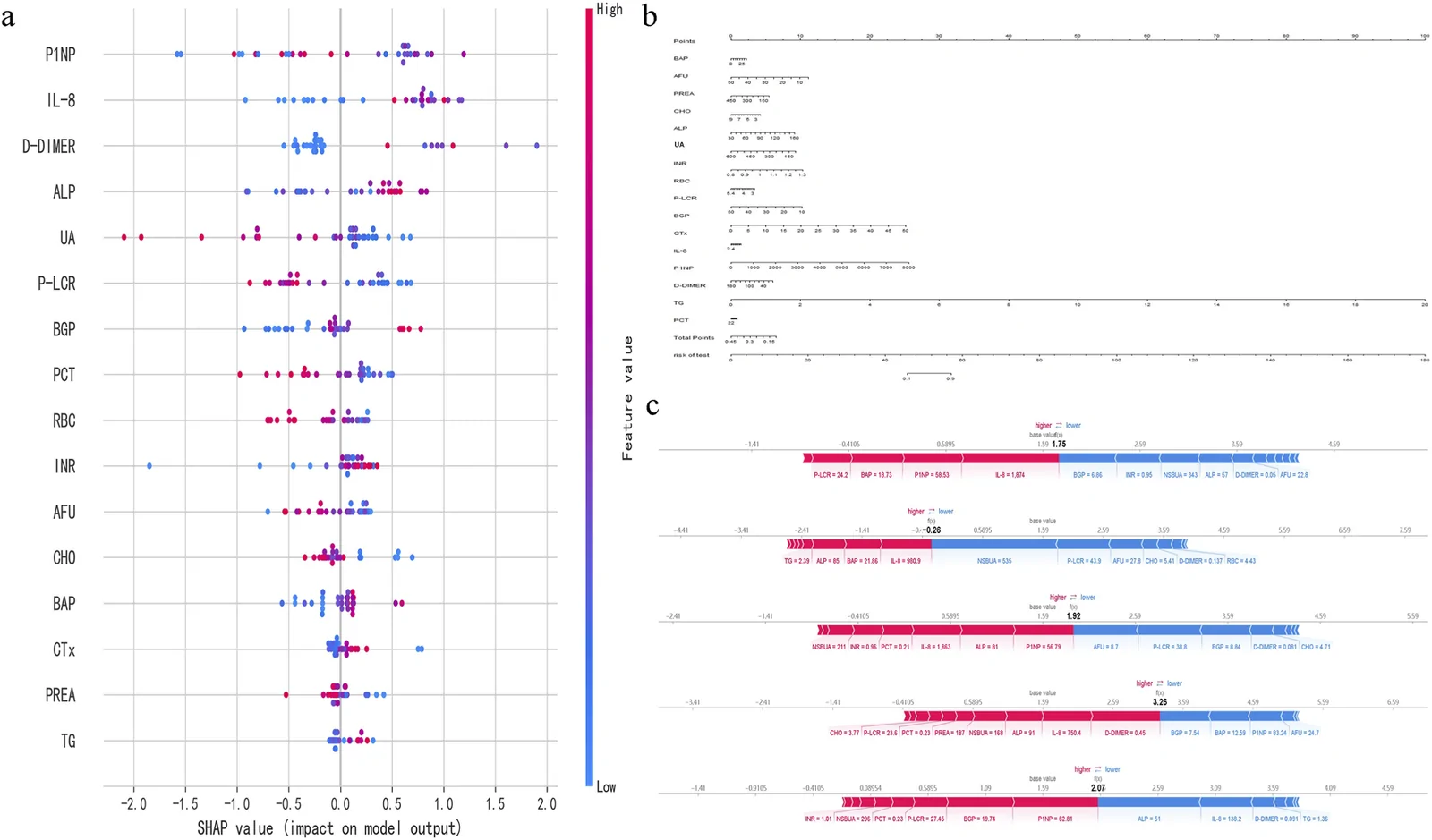
**(a)** SHAP values show the features’ function on predicted value. **(b)** Nomogram for predicting osteoporosis risk. **(c)** SHAP model for five typical predictions.

## Discussion

As an escalating threat to public health, osteoporosis relies on DXA for diagnosis which significantly limited the feasibility for population screening. To address this barrier, several studies have explored the risk factors for osteoporosis and developed predictive models for its early identification. For instance, Kunze et al. conducted a machine learning model to predict 10-year risk of menopause-related osteoporosis, incorporating 14 baseline risk factors including clinical characteristics, laboratory results and imaging data (Zeitlin et al., 2023). Park et al. developed a XGBoost -based prediction model, which included variables such as genetic factor, breastfeeding history, age, residential area, serum albumin and so on (Park et al., 2021). FRAX, recommended by WHO, is currently the mainstream fracture risk assessment tool worldwide. It only relies on simple clinical information such as age, body mass, past fragility fractures, and hormone use to calculate the 10-year fracture probability. Despite the emergence of novel and well-performing methods for bone health assessment, disparities in study quality remain prevalent. On the other hand, many models require an excessive number of input features, such as imaging result, which are not readily accessible in routine clinical practice (Khanagar et al., 2021; Zhang et al., 2024; Hong et al., 2024). These gaps highlight the urgent need in the development, validation, and reporting of AI driven osteoporosis prediction models. Compared with these, the machine learning model constructed in this study integrates 16 blood testing indicators such as IL-8, PINP, CTx, etc., without relying on DXA equipment, and is more suitable for primary screening of large-scale populations at the grassroots level.

Recently, various studies have shown that cytokines participate in precise regulation of immune inflammatory responses in the body through complex signaling networks. In the pathological progression of osteoporosis, some cytokines, especially those involved in the dynamic balance between osteoclasts and osteoblasts, play a key role in bone remodeling (Soh et al., 2023; Wojdasiewicz et al., 2018). These cytokines mainly include members of the tumor necrosis factor family, interleukin family, and growth factors, which together regulate the differentiation, activation, and function of osteoclasts and osteoblasts. Of particular importance, the RANKL/RANK/OPG signaling axis serves as the core regulatory hub of the cytokine network and signaling pathway, playing an irreplaceable role in maintaining bone metabolism balance (Weitzmann, 2017; McLean, 2009). To further investigate the role of cytokines in osteoporosis, we selected 80 representative samples (40 osteoporosis patients and 40 healthy controls) for comparative analysis of cytokine levels. Consistent with the above mechanism insights, our results showed significant differences (p < 0.05) in IL-8 and IL-18 levels between the two groups. These findings confirm that IL-8 and IL-18 are closely related to the pathogenesis of osteoporosis and support their practicality as potential risk factors for predicting osteoporosis. The production and secretion of interleukin-8 (IL-8) are tightly regulated by MAPK signaling pathway. Its primary biological function is to mediates the chemotaxis and activate of neutrophils and recruits T cells and eosinophils to the site of inflammation, thereby amplifying the local inflammatory response (Al-Lami and Al-Hilfy, 2025; Zhu H. et al., 2023; Kany et al., 2019). In clinical practice, as an inflammatory factor, IL-8 can directly act on monocytes/osteoclast precursor cells; On the other hand, IL-8 participates in stimulating osteoblasts to further secrete RANKL, reduce OPG, increase the RANKL/OPG ratio, thereby promoting osteoclast differentiation, enhancing bone resorption, disrupting the dynamic balance of osteoblasts and osteoclasts, and exacerbating bone loss. IL-18 acts as a downstream effector of the NLRP3 inflammasome caspase 1 pyroptosis pathway. Activated NLRP3 inflammasome increases IL-18 levels, drives inflammatory bone damage, and participates in the pathological process of osteoporosis.

In addition to cytokines, characteristics and laboratory parameters were also incorporated to identify key factors distinguishing osteoporosis patients from healthy controls. Initially, we employed various analytical approaches and validation strategies to develop and evaluate a prediction model for osteoporosis. This model was ultimately built using 16 variables, including BGP, CTx, IL-8, PINP, D-DIMER, AFU, TG, CHO, ALP, NSBUA, INR, RBC, P-LCR, PCT, BAP. Based on these identified key factors, we employed 11 machine learning algorithms to build predictive models and evaluated their performance in predicting the risk of osteoporosis. Among these models, Gradient Boost showed the best performance with an AUC value of 0.91, accuracy of 0.81, F1 score of 0.80, and a relatively balanced specificity and sensitivity. Subsequently, the SHAP value indicated the magnitude of which features influence the prediction. A larger positive value indicates the feature increase the probability of predicting osteoporosis, while a large negative value indicates the feature substantially reduce this prediction probability. As illustrated in Figure 8c, the SHAP value visualization for five typical samples intuitively demonstrates the direction and strength of each feature contribution to the osteoporosis prediction for individual samples. For each sample, the model first sets a base value. Then, by summing the positive and negative contributions of all features, the final prediction value is obtained. Compare the final value with the base value to indicate whether the participate was a high risk of osteoporosis or low. We also developed a nomogram that illustrates the importance of each independent risk factor in the predictive model.

Consistent with the finding of previous research, biochemical markers of bone metabolism are widely used in clinical practice for diagnosis, monitoring, and risk stratification of osteoporosis. Among them, BGP is a unique protein synthesized by odentinocytes and osteoblasts (Liu et al., 2023; Ismail et al., 1986). As a major non-collagenous protein secreted by these cells, BGP is currently recognized as a biomarker of the bone formation rate. Notably, this study confirmed that BGP could predict osteoporosis fracture risk, rendering it high valuable for the early prevention of fractures (Liang et al., 2013; Mao et al., 2025). In addition, ALP and PINP are also considered as well established biomarkers of bone formation: ALP plays an important role in bone mineralization, while PINP, a byproduct of type I collagen synthesis, directly mirrors the rate of new bone formation (Bertoldo et al., 2024; Zhu Z. et al., 2023). In contrast, CTx is a key biomarker of bone resorption as it is released during the degradation of mature type I collagen by osteoclasts, specifically indicating osteoclast activity and bone resorption rate (Manriquez et al., 2023; Bhattoa et al., 2025). Consistent with these known functions, our study identified BGP, PINP, ALP, and CTx as important predictive variables, further verifying their clinical practicality in the assessment of osteoporosis.

In addition to bone metabolism biomarkers, previous research reports have shown a positive correlation between bone mass stage and PT, INR, and D-dimer in adults over 50 years old in China (Wang et al., 2024). It is worth noting that D-dimer is significantly negatively correlated with BMD in the Ward’s triangle, which refers to the radiographic area located at the intersection of primary compression, secondary compression, and primary tension trabeculae in the femoral neck (Naud et al., 2020; Luo et al., 2024). The underlying link between coagulation abnormalities and reduced BMD may be attributed to impaired bone microarchitecture: for instance, in patients with diabetes and vascular complications, compromised nutrient delivery to bone tissue and disrupted bone remodeling processes have been shown to accelerate the deterioration of trabecular structure, that could also contribute to the observed correlation between D-dimer and Ward’s triangle BMD in the elderly population. Consistent with these findings, our study also identified coagulation-related indicators as a key predictive variable for osteoporosis, further supporting the potential crosstalk between the coagulation system and bone metabolism.

Dyslipidemia are key driving factors for oxidative stress and inflammation. It has a direct adverse effect on bone health by shifting the balance of bone marrow stromal cells towards adipogenic differentiation and away from osteogenic differentiation. Bone marrow obesity can also secrete pro-inflammatory adipokines, further inhibiting bone formation, promoting osteoclast mediated bone resorption, and ultimately weakening the overall bone structure (Carnovali et al., 2019; Liu et al., 2024). The harmful effects of UA on bone health are mainly attributed to two key processes: first, intracellular oxidative stress generated during UA degradation, and second, UA induced accumulation of pro-inflammatory factors. These processes impair bone homeostasis by inhibiting osteoblast mediated bone formation while promoting osteoclast driven bone resorption (Chang et al., 2024; Tao et al., 2021). In addition, existing studies have shown that UA can inhibit the expression of 1α-hydroxylase in renal proximal tubular cells. Due to the fact that 1α - hydroxylase is a key enzyme in activating vitamin D, its reduction further disrupts the normal bone remodeling process, ultimately increasing the risk of osteoporotic fractures (Cao et al., 2018; Cheng et al., 2019).

According to reports, inflammatory markers from whole blood, such as neutrophil lymphocyte monocyte count and PDW, PCT, ESR, and CRP levels, are negatively correlated with BMD values (Armignacco et al., 2022; Teng et al., 2013). Specifically, as BMD values and t-scores decrease, the level of neutrophil-to-lymphocyte ratio, platelet-to-lymphocyte ratio, monocyte-to-lymphocyte ratio are consistently elevated. This negative correlation is consistent with the recognized link between inflammation and impaired bone homeostasis. The elevation of neutrophils and monocytes reflects a pro-inflammatory systemic state, which can promote the differentiation and activity of osteoclasts. In contrast, lymphocytes play an anti-inflammatory role and support bone formation. Therefore, the relative decrease in lymphocytes further exacerbates the inflammatory imbalance, accelerates bone loss, and reduces BMD (Guo et al., 2025; Ding et al., 2022).

Although the potential mechanisms linking these factors to osteoporosis have not been fully elucidated, the strong clinical relevance provides potential ideas for the development of predictive models, enhancing the clinical value of the conclusions. It is important to emphasize that the predictive model we have developed is designed to provide an auxiliary tool for clinical decision-making. This research still has some limitations that need to be acknowledged. Firstly, this study utilized retrospective data without conducting subgroup analysis stratified by age and menopausal status. In addition, the lack of smoking, exercise, and calcium intake makes it difficult for us to evaluate the predictive ability of the model in different subgroups and limits the representativeness of the study population, which may introduce potential biases. Therefore, there was no synchronous calculation of FRAX risk values in this queue to complete the horizontal efficacy comparison. Secondly, this study was conducted in a single institution with a limited sample size, which resulted in a certain degree of overfitting in the training set model. In the future, multi center large-scale research is needed to verify the reliability of model performance. Thirdly, although we have identified significant associations between coagulation function, lipids, inflammation, and osteoporosis, the underlying molecular and cellular mechanisms driving these associations are still not fully elucidated. Therefore, it is needed to develop further studies to analyze the causal relationships and mechanistic connections between these factors and the pathogenesis of osteoporosis.

## Conclusion

In this study, a machine learning based osteoporosis prediction model was successfully developed, which demonstrated robust performance in clinical applications. By integrating 16 accessible predictive factors, the model avoids the reliance on initial risk assessment of DXA, which helps to implement osteoporosis screening more widely and improves the universality of early risk identification for high-risk populations. Over the long term, this enhanced screening efficiency is expected to provide timely intervention for high-risk populations of osteoporosis, ultimately helping to reduce the incidence of osteoporotic fractures and improve the skeletal health of the population.

## Data Availability

The original contributions presented in the study are included in the article/Supplementary Material, further inquiries can be directed to the corresponding authors.
