# Supplementary material for: Development and evaluation of a machine learning based risk prediction model for osteoporosis
Source: Front Mol Biosci. 2026 Sep 24;13:1939155. doi: 10.3389/fmolb.2026.1939155 (PMC13645573; doi:10.3389/fmolb.2026.1939155)
Supplement: Supplementary file 1 [file Supplementaryfile1.docx]

Figure S1 The differences of selected features between health group and osteoporosis group （Statistical significance: * P < 0.05, ** P < 0.01, *** P < 0.001）

Abbreviations

| BMD | Bone mineral density |
| --- | --- |
| DXA | Dual-energy X-ray absorptiometry |
| BMI | Body Mass Index |
| IFNα, IFNγ | Interferon α, Interferon γ |
| IL-10, IL-17F, IL-1, IL-18, IL-22, IL-2R, IL-2, IL-17A, IL-8, IL-4 | Interleukin-10,  Interleukin-17F,  Interleukin-1,  Interleukin-18, Interleukin-22,  Interleukin-2R,  Interleukin-2,  Interleukin-17A,  Interleukin-8,  Interleukin-4 |
| XGBoost | EXtreme Gradient Boosting |
| ANN | Artificial Neural Network |
| KNN | K-Nearest Neighbors |
| SVM | Support Vector Machine |
| LightGBM | Light Gradient Boosting Machine |
| ROC | Receiver Operating Characteristic Curve |
| AUC | Area Under the ROC Curve |
| BGP | Bone Gla Protein |
| CTx | C-telopeptide of Type Ⅰ Collagen |
| PINP | N-terminal Propeptide of Type Ⅰ Procollagen |
| AFU | Alpha-L-Fucosidase |
| CHE | Cholinesterase |
| TG | Triglyceride |
| CHO | Cholesterol |
| ALP | Alkaline Phosphatase |
| UA | Uric Acid |
| INR | International Normalized Ratio |
| PTA | Prothrombin Time Activity |
| PT | Prothrombin Time |
| RBC | Red Blood Cell |
| P-LCR | Platelet-Large Cell Ratio |
| BASO% | Basophil Percentage |
| EO# | Eosinophil Count |
| HGB | Hemoglobin |
| PDW | Platelet Distribution Width |
| MPV | Mean Platelet Volume |
| PCT | Procalcitonin |
| BAP | Bone-Specific Alkaline Phosphatase |
| LDL | Low-Density Lipoprotein |
| PR curve | Precision-Recall Curve |
| SHAP | SHapley Additive exPlanations |
| MAPK | Mitogen activated protein kinase |
